# Objective assessment of alcohol consumption in early pregnancy using phosphatidylethanol: a cross‐sectional study

**DOI:** 10.1186/s12884-021-03804-7

**Published:** 2021-04-30

**Authors:** Leonieke J. Breunis, Sophie Wassenaar, Barbara J. Sibbles, Ab A. Aaldriks, Hilmar H. Bijma, Eric A.P. Steegers, Birgit C.P. Koch

**Affiliations:** 1 Department of Obstetrics and Gynecology, Erasmus MC Sophia Children’s Hospital, University Medical Center Rotterdam, Wytemaweg 80, 3015 CN Rotterdam, the Netherlands; 2 Department of Hospital Pharmacy, Erasmus MC, University Medical Center Rotterdam, Wytemaweg 80, 3015 CN Rotterdam, the Netherlands; 3 Department of Pediatrics, Erasmus MC Sophia Children’s Hospital, University Medical Center Rotterdam, Wytemaweg 80, 3015 CN Rotterdam, the Netherlands; 4 Department of Psychiatry, Reinier de Graaf Hospital, Reinier de Graafweg 5, 2625 AD Delft, the Netherlands

**Keywords:** Alcohol drinking, Biomarkers, Cross‐section studies, Fetal alcohol spectrum disorders, Netherlands, Phosphatidylethanol, Pregnancy, Prevalence

## Abstract

**Background:**

Alcohol consumption during pregnancy is associated with major birth defects and developmental disabilities. Questionnaires concerning alcohol consumption during pregnancy underestimate alcohol use while the use of a reliable and objective biomarker for alcohol consumption enables more accurate screening. Phosphatidylethanol can detect low levels of alcohol consumption in the previous two weeks. In this study we aimed to biochemically assess the prevalence of alcohol consumption during early pregnancy using phosphatidylethanol in blood and compare this with self-reported alcohol consumption.

**Methods:**

To evaluate biochemically assessed prevalence of alcohol consumption during early pregnancy using phosphatidylethanol levels, we conducted a prospective, cross-sectional, single center study in the largest tertiary hospital of the Netherlands. All adult pregnant women who were under the care of the obstetric department of the Erasmus MC and who underwent routine blood testing at a gestational age of less than 15 weeks were eligible. No specified informed consent was needed.

**Results:**

The study was conducted between September 2016 and October 2017. In total, we received 1,002 residual samples of 992 women. After applying in- and exclusion criteria we analyzed 684 samples. Mean gestational age of all included women was 10.3 weeks (SD 1.9). Of these women, 36 (5.3 %) tested positive for phosphatidylethanol, indicating alcohol consumption in the previous two weeks. Of women with a positive phosphatidylethanol test, 89 % (n = 32) did not express alcohol consumption to their obstetric care provider.

**Conclusions:**

One in nineteen women consumed alcohol during early pregnancy with a high percentage not reporting this use to their obstetric care provider. Questioning alcohol consumption by an obstetric care provider did not successfully identify (hazardous) alcohol consumption. Routine screening with phosphatidylethanol in maternal blood can be of added value to identify women who consume alcohol during pregnancy.

## Background

Fetal Alcohol Spectrum Disorders (FASD) are characterized by a combination of specific facial features, growth deficiency, and neurobehavioral impairment after prenatal alcohol exposure (PAE) [1]. The estimated global prevalence of FASD is 2.3 % [2]. There are no FASD prevalence data available of the Netherlands [2]. Heavy alcohol drinking is considered most harmful to the developing fetus and the first trimester seems to be the most vulnerable period [3–5]. Any amount of alcohol consumption could influence fetal development as prolonged effect of alcohol, due to slower elimination and accumulation in the amniotic fluid (which the fetus then swallows again), occurs [6]. Recently, is was shown that even low levels of maternal alcohol consumption are associated with changes in offspring brain development [7]. Some studies report an increased risk of spontaneous miscarriages and preterm birth, decreased embryonic growth and birth weight, and deviant psychomotor and mental development, while other studies report no adverse health outcomes from small to moderate use of alcohol during pregnancy [4, 5, 8–10]. The American Academy of Pediatrics therefore emphasizes that there is no pregnancy trimester or amount of alcohol consumption that can be considered safe during pregnancy, and the British Medical Association advises complete abstinence during pregnancy [11, 12]. Although the same advice applies in the Netherlands, [13] 4 % of Dutch pregnant women reported consuming alcohol while knowing to be pregnant in 2018, 82 % of these women reported only a few sips [14].

Most studies on PAE depend on maternal self-reported measures, including survey methods and standardized questionnaires. Self-reported measures have been shown to underestimate alcohol use between four and thirteen times, [15] with reasons for doing so including social stigma and difficulties in recalling drinking patterns, amongst others [3, 15, 16]. Lack of disclosing alcohol consumption may lead to inadequate support for mother and (unborn) child, persistent alcohol use during pregnancy, fetal brain damage, and delay in the diagnosis of FASD. A delay in the diagnosis of FASD results in lack of developmental and social support for affected children and their families. This support is of great importance in attaining the best possible outcome for these children, such as a four-fold decrease in the chance of alcohol- and drug related problems compared to children with delayed diagnosis of FASD who are being reared in unstable environments [17].

Hence, objective biomarkers are important in identifying alcohol use, and subsequently PAE, during pregnancy. Unfortunately, many biomarkers for alcohol exposure during pregnancy have limitations because they either assess only short term alcohol consumption (ethanol, ethyl glucuronide), detect only high levels of alcohol exposure (carbohydrate deficient transferrin, microRNA), have a low sensitivity or specificity (mean corpuscular volume, gamma-glutamyltransferase, aspartate aminotransferase/alanine aminotransferase ratio), or can only be performed after birth (Fatty Acid Ethyl Esters) [18, 19]. Phosphatidylethanol (PEth) is the only biomarker that can identify even low levels of alcohol consumption over a longer period of time [18, 20, 21]. PEth is a group of abnormal phospholipids formed in cell membranes of red blood cells exclusively in the presence of ethanol [22]. As a result PEth is only detectable after alcohol consumption, even after a single unit, [23] and the presence of PEth is therefore indicative for alcohol consumption. Differences in half-life of PEth, ranging from 1 to 13 days in healthy non-pregnant participants, have been described due to intra-patient variability [21, 23, 24]. With a mean half-life of four days, this biomarker remains detectable for two weeks after consumption a single unit of alcohol and for a longer period after larger amounts of alcohol intake [25]. A high value of PEth is indicative for heavy use of alcohol [18].

The aim of this study was to determine the prevalence of PAE in early pregnancy, before fifteen weeks of gestation, in an urban university hospital in the Netherlands, by measuring PEth in maternal blood. In addition, we aimed to compare this objective assessment with self-reported alcohol use.

## Methods

### Design

This is a prospective cross-sectional study.

### Setting and participants

The study was conducted between September 2016 and October 2017 at the Erasmus MC, a tertiary hospital in an urban area in the Netherlands. In the Netherlands, approximately 90 % of pregnancies are planned [26]. Most pregnant women receive antenatal care from community midwives. However, women with medium and high risk pregnancies receive antenatal care in hospitals by clinical midwives or gynecologists (in training). Women who visit the Erasmus MC for their first antenatal visit generally receive an appointment several weeks after being referred to the outpatient clinic, but preferably before the 16th week of gestation. In addition, all pregnant women receive an ultrasound around twenty weeks of gestation. If this ultrasound shows abnormalities, women are also referred to a hospital. The same applies for women who develop complications (e.g. pre-eclampsia) during their pregnancy. During the first prenatal visit at the hospital, all pregnant women routinely undergo blood sampling. All pregnant women referred to the outpatient obstetric clinic of the Erasmus MC for pregnancy care who underwent routine pregnancy blood sampling before a gestational age of 15 weeks, were eligible. Women under 18 years of age, with an unknown gestational age, and those who did not consent to the use of residual material (e.g. residual blood or placenta) were excluded.

Prior to this study, we conducted a pilot study in which we actively asked consent to all eligible women to screen for alcohol consumption in their residual blood. This pilot study was performed to assess the percentage of women declining consent. A considerable number of women, 16 % (18 out of 113), refused participation. Due to the probability this causes an important selection bias, we decided, together with the Medical Ethical Committee of the Erasmus MC, not to continue that research but to conduct a new study. For this current study, no specified informed consent was asked. For this study, women were informed through pamphlets in the waiting room and were offered to opt-out for the use of residual material. Women who opted-out were also excluded. Moreover, if multiple blood samples of one patient were collected, we only used the first sample.

### Data collection

#### Patient characteristics

We extracted maternal age, gravidity, parity, country of birth, gestational age, as well as self-reported use of tobacco, alcohol or illicit drugs, from the patient records. During the first visit, the use of tobacco, alcohol or illicit drugs is verbally asked by the doctor according to standard questions in the patient records (“Did you use alcohol during this pregnancy? And are you currently using?”).

#### PEth test

All pregnant women have blood drawn at their first visit as part of general screening (e.g. blood type, hemoglobin, infectious diseases). The residual blood was used for this study. The residual blood was frozen at -80 degrees Celsius for a maximum of six months. We measured PEth in the residual whole blood. The PEth test has been validated in our ISO accredited laboratory according to Food and Drug Administration and European Medicines Agency validation [22, 27]. Correlation coefficients were higher than 0.995 for all three compounds. Intraday and interday inaccuracies were < 15 % for all analytes in the established linear range. Intraday and interday imprecision were < 15 % for all analytes. Sample stability at -80 °C was one year. Extracts were stable for one day in the autosampler and two days at 2–8 °C in a closed Eppendorf tube. Samples were tested after three freeze-thaw cycles and considered stable. The total PEth concentration is based on the sum of 1-palmitoyl-2-oleoyl-sn-glycero-3-phosphoethanol (POPEth, also referred to as PE 16:0/18:1), 1-palmitoyl-2-linoleoyl-sn-glycero-3-phosphoethanol (PLPEth, also referred to as PE 16:0/18:2) and 1,2-dioleoyl-sn-glycero-3-phosphoethanol (DOPEth, also referred to as PE 18:1/18:1), all in µg/L. The PEth test was classified as positive if at least one of these values was above the limit of detection (LOD). The lower limit of quantification (LLOQ) and LOD of POPEth and PLPEth were 6.0 and 2.0 µg/L, respectively. The LLOQ and LOD of DOPEth were 3.0 and 2.0 µg/L respectively. Healthcare providers did not receive the results of the PEth test.

### Data analysis

Before data collection we performed a sample size calculation. When assuming a prevalence of 1 %, with a 95 % confidence interval (CI) and a precision of 0.5 % (meaning a 95 % CI upper limit of prevalence plus precision and lower limit 95 % CI of prevalence minus precision), 1,521 women were needed. If a prevalence of 2 % (95 % confidence interval, precision 1 %) is assumed, 753 women had to be included in the study. About 2200 new pregnant women visit the department every year. That is why we assumed that including 1000 new pregnant women during a period of approximately 32 weeks was feasible. Due to slow inclusion, this period was prolonged. We did not expect exclusions.

Associations between positive PEth tests and several patient characteristics were explored using standard descriptive statistics such as mean and median and analyzed using univariate logistic regression analysis. Data were analyzed using IBM SPSS statistics 25.

### Ethical approval

The study was reviewed and agreed upon by the Medical Ethics Review Committee of the Erasmus MC (protocol ID NL53549.078.15). The need for specified informed consent was waived.

## Results

### Study population

**Fig. 1:**
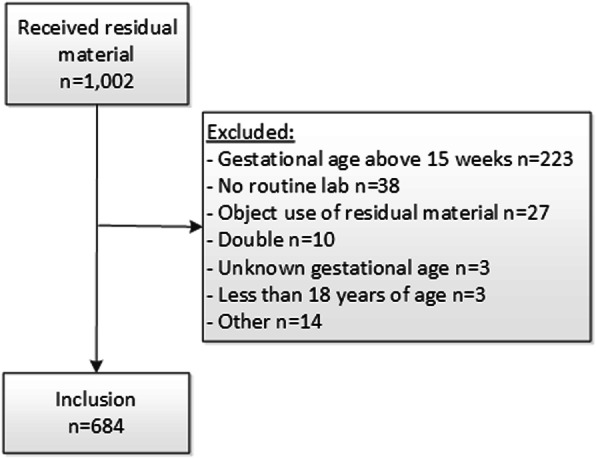
Selection of the study population. Shows the number of samples received, how many samples were excluded from analysis and how many samples were included

Residual blood of all women who visited the outpatient clinic for the first time this pregnancy and who underwent routine blood sampling was available (*n* = 954). 270 women were excluded based on the in- and exclusion criteria, and the unique samples of 684 women were analyzed (Fig. 1). Main reasons for exclusion were that women were more than 15 weeks pregnant (*n* = 223) or that women did not consent to the use of residual material (*n* = 27). The mean age was 31.7 years (SD 4.9), 56.7 % were of Dutch origin. The mean week of gestation was 10.3 weeks (SD 1.9). Of the participants, 22.5 % (*n* = 154) were primigravida and 34.5 % (*n* = 236) were nulliparous. Furthermore, 0.9 % (*n* = 6) of included women reported alcohol consumption.

### Result of the PEth test

Of the 684 included women, 5.3 % (*n* = 36) had a positive PEth test (Table 1). The mean week of gestation of women with a positive PEth test was 9.6 weeks (SD 1.9). Of these women 11 % (*n* = 4) reported alcohol consumption to their obstetric care provider. Women who reported alcohol consumption during pregnancy also had a positive PEth test significantly more often (OR 39.8; 95 % CI 7.0 to 225.5). Of all 36 positive PEth tests, 16 (44.4 %) had at least one value below the LLOQ but above the LOD (meaning that PEth was present but not enough to quantify). Of the women with a negative PEth test (*n* = 648), the mean gestational age was 10.4 weeks (SD 1.9). Two women (0.3 %) reported alcohol consumption despite a negative PEth test.

**Table 1:** Characteristics of the study population^a^

Characteristic	PEth negative ***n***=648	PEth positive ***n***=36	Odds ratio (95% CI)
Age, mean years (SD)	31.6 (4.9)	32.3 (4.9)	
<25	45 (6.9)	3 (8.3)	Ref.
25-29	174 (26.9)	7 (19.4)	0.603 (0.150-2.427)
30-34	252 (38.9)	16 (44.4)	0.952 (0.267-3.402)
35-39	136 (21.0)	8 (22.2)	0.882 (0.224-3.469)
>39	41 (6.3)	2 (5.6)	0.732 (0.116-4.601)
Weeks of gestation, mean (SD)	10.4 (1.9)	9.6 (1.9)	
Gravida, mean (SD)	1.8 (0.4)	1.8 (0.4)	
1	148 (22.8)	6 (16.7)	Ref.
≥2	500 (77.2)	30 (83.3)	1.480 (0.604-3.624)
Parity, mean (SD)	1.7 (0.5)	1.8 (0.4)	
0	229 (35.3)	7 (19.4)	Ref.
≥1	419 (64.7)	29 (80.6)	2.264 (0.977-5.250)
Smoking			
During conception	58 (9.2) ^b^	5 (14.3) ^c^	1.652 (0.617-4.422)
Current	39 (6.1) ^d^	2 (5.6)	0.908 (0.210-3.919)
Birth country^e^			
The Netherlands	476 (74.1)	23 (63.9)	Ref.
Morocco	29 (4.5)	-	-
Turkey	16 (2.5)	-	-
Curaçao	26 (4.0)	3 (8.3)	2.388 (0.673-8.471)
Suriname	14 (2.2)	4 (11.1)	5.913 (1.804-19.386)
Other	81 (12.6)	6 (16.7)	1.533 (0.606-3.881)

*PEth* Phosphatidylethanol

^a^ Data are presented as n(%), unless stated otherwise

^b^ 15 missing

^c^ 1 missing

^d^ 7 missing

^e^ 6 missing in PEth negative group

### Predictors of alcohol consumption

Age, week of gestation, gravida, parity, smoking and country of birth were not significantly associated with a positive PEth test. However, this might be due to the low number of women with a positive PEth test and results are therefore inconclusive.

## Discussion

More unborn children at risk for PAE are identified with the use of a PEth test than discussion of alcohol consumption by the obstetric care provider alone. Because of the low rate of unplanned pregnancies in the Netherlands and the time until the first antenatal visit, we believe the alcohol was consumed while knowing about being pregnant.

Some previous studies compared PEth with self-reported alcohol use [28, 29], the AUDIT questionnaire [30, 31] another biomarker [29, 31] or used a higher treshold for a positive PEth test [31, 32]. Our study is the first to measure PEth in such a large number of pregnant women and without the need for specified informed consent. We concluded more women who consume alcohol are identified with the use of PEth compared to self-report, this is supported by both Raggio et al. and Bracero et al. [28, 29]. The AUDIT questionnaire identifies problematic alcohol consumption, but is not validated in pregnant women and results in this population are mixed [30, 31]. On one hand, it was found that women report alcohol consumption accurately using the AUDIT questionnaire [31], but also that pregnant women with a high AUDIT score tend to have a negative PEth test [30]. The AUDIT questionnaire has not been validated in pregnancy, and we hypothesize that pregnant women in a research setting might feel guilty about their alcohol consumption and therefore give themselves higher scores within the AUDIT questionnaire. Nevertheless, these findings also underline that asking about alcohol consumption does not accurately determine hazardous alcohol consumption during pregnancy and an objective biomarker is needed. In our study only one biomarker was considered, but as suggested in the systematic review of Howlett et al. [33], the combination of biomarkers that screen for alcohol consumption could increase detection of PAE. In addition, our study did not report birth outcomes of the offspring of participating women, whereas Yang et al. [32] did report these outcomes and were able to make conclusions on the effect of alcohol consumption during pregnancy.

Although the majority of guidelines advise on complete abstinence of alcohol during pregnancy, many women report conflicting messages from their healthcare providers on alcohol consumption during pregnancy [34–36]. Moreover, obstetric care providers often only advise complete abstinence once women report alcohol consumption [37]. Bearing in mind that within this study 89% of women with a positive PEth test did not report alcohol consumption, many women do not receive the advice of complete abstinence. Obstetric care providers should keep in mind that many women still consume alcohol during pregnancy and that this should be openly discussed during each consultation. If a PEth test would be part of the routine prenatal screening, it would provide grounds for obstetric care providers to properly discuss alcohol consumption during pregnancy. However, PEth as routine prenatal screening can also cause women to avoid prenatal care and we advocate that when discussing PEth as routine screening, obstetric care providers should also discuss alcohol consumption and offer support and treatment before performing the test.

Even though this study found a prevalence of alcohol consumption of more than 5%, this may only reflect a proportion of the actual PAE. On average, the PEth test indicates alcohol consumption during the previous two weeks, so alcohol use that occurs more than two weeks prior to testing goes undetected. This may lead to underdiagnoses, especially in the case of episodic binge-drinking [3]. Moreover, intra-patient variability concerning half-life of PEth has been described and a few women consuming alcohol can be missed if they eliminate PEth very fast. A potential solution to these problems is the combination of multiple biomarkers, such as PEth and Fatty Acid Ethyl Esters in meconium. In addition, more knowledge on the effects of low levels of alcohol consumption on the fetus is needed so as to provide advice on this topic that is evidence based.

More knowledge is also needed on the cost-effectiveness of routine screening for alcohol use during early pregnancy, as well as on counselling and treatment strategies in the case of alcohol use during pregnancy.

A major strength of this study is that the PEth test was performed on residual material from routine pregnancy testing, specified informed consent was not requested and nearly all women of interest were included, hereby minimizing selection bias. In addition, we included women with a gestational age under 15 weeks who routinely undergo laboratory testing regardless of their health or pregnancy complications also minimizing the risk for selection bias. Alcohol consumption was discussed by the obstetric care provider as done in a normal clinical setting and not as part of research, which increases the relevance and generalizability of this study for clinical practice.

One limitation of this study is that it was done in a tertiary medical center without low risk pregnancies and with much awareness surrounding the importance of periconceptional lifestyle factors, decreasing the generalizability of our results to the general population. The prevalence of alcohol consumption during pregnancy may differ from the general population with low risk pregnancies or without the awareness of the importance of periconceptional lifestyle factors. In addition, because alcohol consumption during pregnancy is associated with an increased risk of miscarriage [8], the prevalence may by higher because some women who consume alcohol already had a miscarriage before visiting the antenatal clinic. A second limitation is that samples were excluded, although we had not taken this into account during the sample size calculation beforehand. Thirdly, we studied associations between positive PEth tests and patient characteristics but the study was not powered to these analyses and the results are inconclusive.

## Conclusions

Despite recommendations on alcohol abstinence, one in nineteen women continued to consume alcohol during early pregnancy. Questioning alcohol consumption by an obstetric care provider did not successfully identify (hazardous) alcohol consumption. Routine screening with phosphatidylethanol in maternal blood can be of added value to identify women who consume alcohol during pregnancy, so targeted counselling and referral for treatment can be initiated.

## Data Availability

The dataset generated and/or analyzed during the current study are available from the corresponding author on reasonable request.

## Abbreviations

CI: Confidence interval
FASD: Fetal Alcohol Spectrum Disorders
LLOQ: Lower limit of quantification
LOD: Limit of detection
PAE: Prenatal alcohol exposure
PEth: Phosphatidylethanol
